# Venous Thromboembolism (VTE) Risk Assessments in Psychiatric Inpatients Audit

**DOI:** 10.1192/bjo.2025.10617

**Published:** 2025-06-20

**Authors:** Rahul Kanabar, Cynthia Onyeanuforo, Arnold Kusi, Shehroz Shakeel

**Affiliations:** Coventry and Warwickshire Partnership NHS Trust, Coventry and Warwickshire, United Kingdom

## Abstract

**Aims:** The aim of this audit was to assess the compliance of Coventry and Warwickshire Partnership NHS Trust (CWPT) with the National Institute for Health and Care Excellence (NICE) venous thromboembolism (VTE) in over-16s guidelines. NICE guidelines recommend that all acute psychiatric patients should be assessed to identify their risk of VTE and bleeding as soon as possible after admission to hospital or by the time of the first consultant review. In addition, NICE guidelines also recommend that all patients admitted to an acute psychiatric ward should be reassessed for risk of VTE and bleeding at the point of consultant review.

**Methods:** All patients admitted to inpatient wards in CWPT are required to have a digital physical health document, which contains a section on VTE risk assessment, completed by the duty doctor. The digital physical health document for all inpatients (n=244) across 16 wards in CWPT were retrospectively reviewed in October 2024. A standardised tool was created to collect data using an adaptation of the NICE VTE guidelines. This tool ensured parallel data was collected for each patient, including whether patients had a VTE risk assessment completed on admission to hospital, whether VTE risk assessments were dated and signed, at what point in time VTE risk assessments were completed following admission, and whether patients had a VTE risk assessment completed at the point of consultant review.

**Results:** 63% (n=153) of patients had a VTE risk assessment completed and documented on admission to hospital, including being signed and dated. 63% (n=154) of patients had a VTE risk assessment completed within 24 hours of admission. 99% (n=242) of patients did not have a VTE risk assessment completed at the point of consultant review. 5% (n=13) of patients had a VTE risk assessment completed without being signed and/or dated. 7% (n=16) of patients had documentation of communication of assessment with a registered mental health nurse.

**Conclusion:** CWPT’s compliance with NICE recommendations for VTE risk assessment was deemed below standard. Recommendations have been made to introduce a VTE risk assessment section into every new doctor’s induction, to ensure they are aware of the importance of completing them and how to complete them appropriately. In addition, if possible, making the VTE risk assessment a required field to submit the physical health aspect of the clerking proforma would aid in increasing compliance rates. A re-audit in 6–12 months is also recommended.

